# Supplementary material for: The Regulatory Roles of Ezh2 in Response to Lipopolysaccharide (LPS) in Macrophages and Mice with Conditional Ezh2 Deletion with LysM-Cre System
Source: Int J Mol Sci. 2023 Mar 10;24(6):5363. doi: 10.3390/ijms24065363 (PMC10049283; doi:10.3390/ijms24065363)
Supplement: Supplementary file 1 [file ijms-24-05363-s001.zip › ijms-2243635-supplementary.pdf]

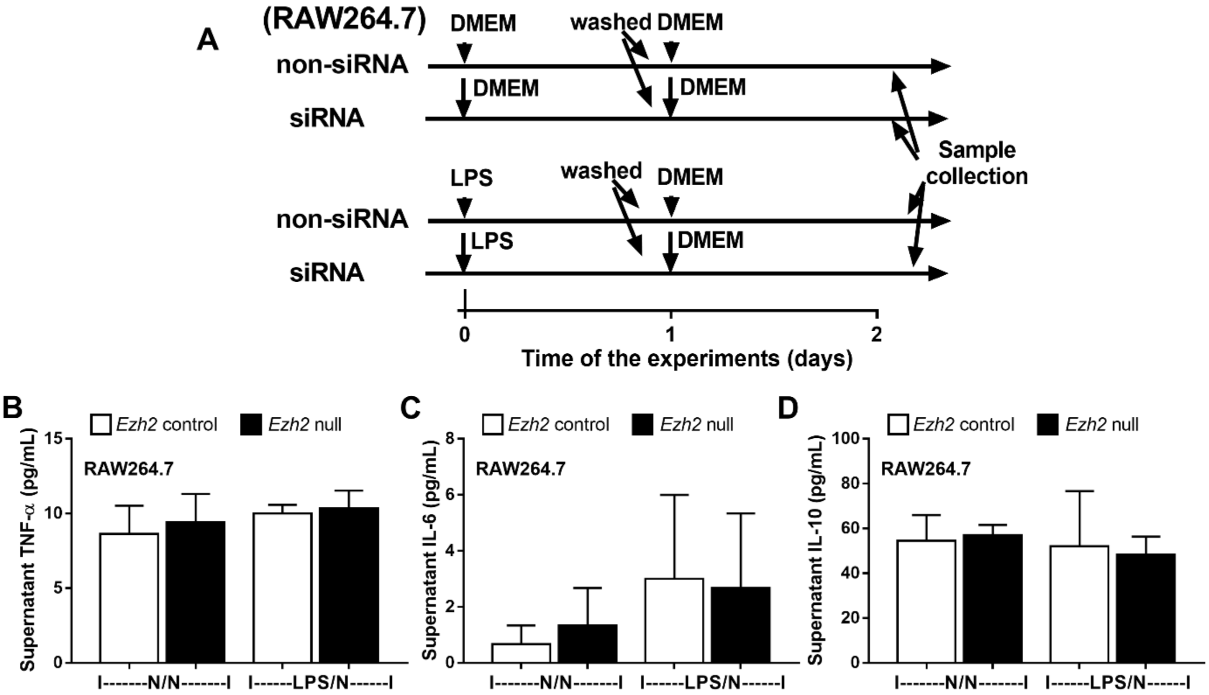

Supplementary Figure S1. The schema of the experiments in murine macrophage cell line (RAW264.7) with the silencing of Ezh2 gene using small interfering RNA (Ezh2 siRNA) or the control siRNA (non-targeting pool siRNA; non-siRNA) and activated by lipopolysaccharide (LPS) followed by the culture media at 24 h later (LPS/N) or control (N/N) using the twice culture media incubation (A) is demonstrated. The characteristics of these macrophages at 2 days of the stimulation as indicated by supernatant cytokines (TNF-α, IL-6, and IL-10) (B-D) are demonstrated. Triplicated independent experiments were performed. Mean ± SEM is presented with the one-way ANOVA followed by Tukey’s analysis (no significant difference among the value).
